# Population Structure of *Staphylococcus aureus* from Remote African Babongo Pygmies

**DOI:** 10.1371/journal.pntd.0001150

**Published:** 2011-05-10

**Authors:** Frieder Schaumburg, Robin Köck, Alexander W. Friedrich, Solange Soulanoudjingar, Ulysse Ateba Ngoa, Christof von Eiff, Saadou Issifou, Peter G. Kremsner, Mathias Herrmann, Georg Peters, Karsten Becker

**Affiliations:** 1 Institute of Medical Microbiology, University of Münster, Münster, Germany; 2 Medical Research Unit, Albert Schweitzer Hospital, Lambaréné, Gabon; 3 Institute of Hygiene, University of Münster, Münster, Germany; 4 Institute of Tropical Medicine, University of Tübingen, Tübingen, Germany; 5 Pfizer Pharma GmbH, Berlin, Germany; 6 Institute of Medical Microbiology and Hygiene, University of Saarland, Homburg, Germany; Mahidol University, Thailand

## Abstract

**Background:**

Pandemic community-acquired methicillin-resistant *Staphylococcus aureus* isolates (CA-MRSA) predominantly encode the Panton-Valentine leukocidin (PVL), which can be associated with severe infections. Reports from non-indigenous Sub-Saharan African populations revealed a high prevalence of PVL-positive isolates. The objective of our study was to investigate the *S. aureus* carriage among a remote indigenous African population and to determine the molecular characteristics of the isolates, particularly those that were PVL-positive.

**Methodology/Principal Findings:**

Nasal *S. aureus* carriage and risk factors of colonization were systematically assessed in remote Gabonese Babongo Pygmies. Susceptibility to antibiotics, possession of toxin-encoding genes (i.e., PVL, enterotoxins, and exfoliative toxins), *S. aureus* protein A (*spa*) types and multi-locus sequence types (MLST) were determined for each isolate. The carriage rate was 33%. No MRSA was detected, 61.8% of the isolates were susceptible to penicillin. Genes encoding PVL (55.9%), enterotoxin B (20.6%), exfoliative toxin D (11.7%) and the epidermal cell differentiation inhibitor B (11.7%) were highly prevalent. Thirteen *spa* types were detected and were associated with 10 STs predominated by ST15, ST30, ST72, ST80, and ST88.

**Conclusions:**

The high prevalence of PVL-positive isolates among Babongo Pygmies demands our attention as PVL can be associated with necrotinzing infection and may increase the risk of severe infections in remote Pygmy populations. Many *S. aureus* isolates from Babongo Pygmies and pandemic CA-MRSA-clones have a common genetic background. Surveillance is needed to control the development of resistance to antibiotic drugs and to assess the impact of the high prevalence of PVL in indigenous populations.

## Author Summary

*Staphylococcus aureus* is a bacterium that colonizes humans worldwide. The anterior nares are its main ecological niche. Carriers of *S. aureus* are at a higher risk of developing invasive infections. Few reports indicated a different clonal structure and profile of virulence factors in *S. aureus* isolates from Sub-Saharan Africa. As there are no data about isolates from remote indigenous African populations, we conducted a cross-sectional survey of *S. aureus* nasal carriage in Gabonese Babongo Pygmies. The isolates were characterized regarding their susceptibility to antibiotic agents, possession of virulence factors and clonal lineage. While similar carriage rates were found in populations of industrialized countries, isolates that encode the genes for the Panton-Valentine leukocidin (PVL) were clearly more prevalent than in European countries. Of interest, many methicillin-susceptible *S. aureus* isolates from Babongo Pygmies showed the same genetic background as pandemic methicillin-resistant *S. aureus* (MRSA) clones. We advocate a surveillance of *S. aureus* in neglected African populations to control the development of resistance to antibiotic drugs with particular respect to MRSA and to assess the impact of the high prevalence of PVL-positive isolates.

## Introduction

Methicillin-resistant *Staphylococcus aureus* (MRSA) has emerged as a community-acquired pathogen in many countries throughout the world (community-acquired MRSA, CA-MRSA). CA-MRSA mostly causes skin or soft-tissue infections as well as deep-seated infections such as necrotizing pneumonia. Predominantly, CA-MRSA encodes the Panton-Valentine Leukocidin (PVL), a *S. aureus* exotoxin that induces lysis of monocytes and neutrophil granulocytes [1]. In African countries, the occurrence of CA-MRSA has been reported previously from Egypt [2], Mali [3], Algeria [4] and Nigeria [5].

Interestingly, population analysis of global methicillin-susceptible *S. aureus* (MSSA) isolates associated with PVL have recently indicated that PVL-positive MSSA and MRSA are phylogenetically related based on molecular epidemiological profiles and are dynamically interrelating [6]. Moreover, it was shown, that PVL-positive MSSA are a likely reservoir for the development of PVL-positive MRSA [6] via integration of *Staphylococcus* cassette chromosome *mec* (SCC*mec*) elements including the *mecA* gene conferring methicillin resistance. Indeed, it is striking that reports from African countries have recently described a high prevalence of PVL-positive MSSA isolates in Nigeria [7] and Mali [3] and have supported the hypothesis that at least one common European MRSA clone associated with PVL (sequence type ST152 according to multilocus sequence typing (MLST)) could originate from African MSSA clones [3]. Interestingly, a study on *S. aureus* colonization in Wayampi Amerindians in French Guiana revealed a predominance of ST1223 which is highly divergent from other global STs [8]. Ruimy et al. hypothesize, that this association of highly divergent clones in an isolated remote population may reflect the co-evolution of humans and *S. aureus* as well as human migration [8], [9].

Consequently, we raise the question, whether the “out-of-Africa” hypothesis, as shown for *Helicobacter pylori* [10], might also be true for PVL-positive *S. aureus* clones now emerging across the globe. To address this question, we aimed to collect systematically *S. aureus* isolates independent from the healthcare setting, which is associated with the dissemination of isolates adapted to the specific selection pressure of the hospital environment. Therefore, we performed a cross-sectional *S. aureus* carrier study among the indigenous Pygmy population in Gabon. One to five percent of the Gabonese population is comprised by Pygmy hunter-gatherers. Almost 50% belong to the Babongo tribe, most of them are living in Waka National Parc, Central Gabon.

## Materials and Methods

### Ethics statement

Ethical clearance was obtained from our institutional review board (IRB, “Comité d'Éthique Régional Indépendant de Lambaréné”, Lambaréné, Gabon, protocol number: CERIL 15–09). As the majority of Babongos are illiterate and mainly speak the tribal language, we involved a local interpreter to provide detailed information about the study and to obtain a documented oral informed consent. We prepared a short written summary in French that described the information presented to the Pygmies. This document was signed or finger-printed by the participant, the researcher and a witness who spoke French and Babongo. The IRB approved the use of documented oral informed consent.

### Study population

A cross-sectional survey of *S. aureus* nasal carriage in Babongo Pygmies was conducted as part of the German-African network on staphylococci and staphylococcal diseases (DFG PAK 296) and took place in the Ikobé region, Central Gabon in November 2009.

All Babongo or mixed Babongo-Bantu inhabitants of the Ikobé region were included if they provided a documented informed consent. Exclusion criteria were (i) infections of nostrils and (ii) a purulent rhinitis. Demographic data (self reported age, height, weight, sex and ethnic group) were recorded for each subject. Travel habits since birth and daily activities were recorded to assess risk factors for *S. aureus* carriage. Global positioning data of each village were taken by GPS-device (Garmin76 csx).

### Bacterial isolates

Nasal swabs were stored in cool boxes and inoculated on SAID agar plates (bioMérieux, Marcy l'Etoile, France) and Columbia blood agar plates in the laboratory facilities of the Medical Research Unit, Lambaréné within four days after sampling. Presumptive *S. aureus* isolates were identified by colony characteristics, catalase and latex agglutination test (Pastorex Staph-Plus, Bio-Rad Laboratories, Marnes-la-Coquette, France). Species identification and antibiotic susceptibility testing were performed by Vitek 2 automated systems (bioMérieux, Marcy l'Etoile, France). Molecular confirmation of *S. aureus* and determination of methicillin-resistance were performed as described [11]. To confirm susceptibility to penicillin, a *blaZ* PCR targeting the *S. aureus* penicillinase was performed additionally [12].

### Virulence factors, capsular polysaccharides and *agr* subtypes

Panton-Valentine leukocidin (PVL) encoding genes (*luk*S-PV, *luk*F-PV) were detected [13]. Staphylococcal pyrogenic toxin superantigens (PTSAgs) were analyzed by detecting toxic shock syndrome toxin (TSST-1) encoding genes (*tst*) and the enterotoxins (*sea, seb, sec, sed, see, seg, seh, sei* and *sej*) [14], [15]. Exfoliative toxins (*eta, etb* and *etd*) and genes encoding members of the epidermal cell differentiation inhibitor (*edin-A, edin-B* and *edin-C*) were detected by gene amplification [14]–[17].

Capsular polysaccharide types 5 and 8 and accessory gene regulator subtypes (*agr* I–IV) were identified by multiplex PCR approaches [13], [18].

### Genotyping

*S. aureus* isolates were typed based on sequencing of the hypervariable region of the *S. aureus* protein A gene (*spa*), *spa* types were assigned on the Ridom SpaServer (http://spaserver.ridom.de) curated by the SeqNet.org initiative [19]. Multilocus sequence typing (MLST) was carried out for each isolate [20]. Relatedness in allelic profiles was assessed using eBURST (version 3, http://eburst.mlst.net). To affiliate the *S. aureus* sequence types (STs) of the Pygmy population to known clonal complexes (CC), we compared our dataset with the whole MLST database of *S. aureus* using the stringent group definition of a minimum of 6/7 shared alleles.

### Statistics

Proportions of categorical variables were tested using Chi-square test and Fisher's exact test, where appropriate. Odds-ratio and the 95% confidence intervals were calculated to test for associations. The level of significance was α = 5%. All analyses were performed using the software “R” (http://cran.r-project.org, Version: 2.10.1) and package “epicalc”.

## Results

### Study population

**Figure 1:**
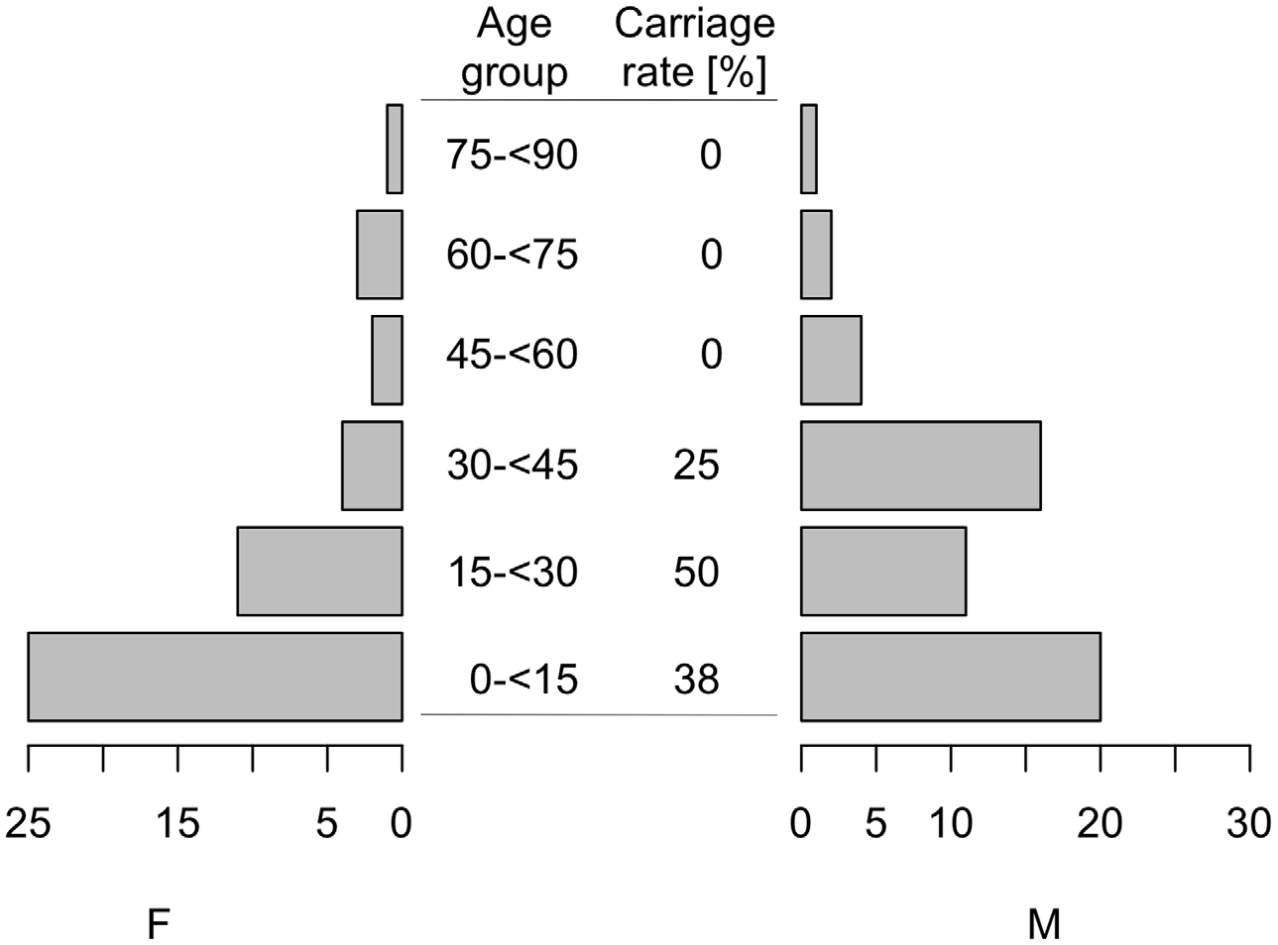
Pagoda-shaped population pyramid of the study population and S. aureus carriage rates (%) in different age groups. X-axis indicates the percentage of male (M) and female (F) participants of the whole study population, y-axis represents age groups and the carriage rate of *S. aureus* in the respective age group.

Nasal swabs were obtained from 99 Babongo Pygmies and 1 Babongo-Mitsogho living in the Ikobé region, Gabon. Study participants came from five camp-like villages (GPS coordinates in brackets) made up of about six to ten huts: “Village Tranquille” (S1°02.392′; E11°03.744′), “Tsibanga” (S1°02.577′; E11°05.661′), “Ossimba” (S1°02.433′; E11°04.655′), “Ndougou” (S1°02.316′; E11°04.417′), “Soga” (S1°03.020′; E11°10.963′) and “Egouba” (S1°01.638′; E11°08.123′). All villagers who had been in the villages during our visit met the inclusion criteria (n = 103). Three persons from Egouba refused to participate. The age distribution of participants showed a pagoda-shaped population pyramid with 45% of participants being ≤15 years old and 13% being ≥45 years old (Figure 1). Overall, 46% of participants were female. Of all participants, 65% have travelled at least to one of the nine capital cities of Gabon since birth. Twenty-six percent (n = 26) of the population had been hospitalized previously in dispensaries or primary-care hospitals (Table 1).

**Table 1:** Demographic characteristics of the Babongo population in the Ikobé region, Gabon.

Demographic data	Village Tranquille	Tsibanga	Ossimba	Ndougou	Soga	Egouba	Total
No. participants	46	15	19	5	13	2	100
Mean age in years (range)	21.7 (0.17–84)	23.0 (2–47)	22.4 (0.08–70)	31 (3–49)	30.8 (6–49)	37.5 (35–40)	23.8 (0.08–84)
Female (%)	50	40	47.4	20	53.8	0	46
Mean weight (kg) ±SD	30.09±16.67	39.49±19.09	34.47±18.82	46.66±25.47	39.45±11.25	48.35±1.91	34.74±17.64
Mean height (m) ±SD	1.19±0.33	1.38±0.26	1.28±0.36	1.28±0.27	1.43±0.16	1.53±0.04	1.28±0.31
Travelling since birth (%)	73.9	53.3	57.9	40	61.5	100	65
Health care contact (%)	19.6	33.3	42,1	40	7.7	50	26
No. *S. aureus* carriers (%)	21 (45.7)	2 (13.3)	5 (26.3)	0	4 (30.8)	1 (50)	33
Penicillin resistance (%)	33.3	50.0	40.0	NA^a^	40.0	0	35.3
Tetracycline resistance (%)	9.5	0	0	NA^a^	0	0	5.8
Trimethoprim-sulfamethoxazole resistance (%)	19.4	0	0	NA^a^	0	0	11.8
*spa* type (%)	t084 (19.1), t148 (9.5), t159 (4.8), t1848 (33.3), t186 (14.3), t1931 (4.8), t311 (4.8), t5941 (9.6)	t148 (50), t570 (50)	t148 (20), t186 (20), t189 (20), t1931 (20), t6020 (20)	NA^a^	t127 (20), t189 (20), t5941 (40), t6025 (20)	t1848 (100)	

a not applicable.

### *S. aureus* carriage and antimicrobial resistance

Culture of 100 nasal swabs identified 34 *S. aureus* isolates. The carriage rate was 33%. From one participant, two phenotypically different *S. aureus* isolates were isolated (white colonies with β-hemolysis and yellow-white colonies with faint β-hemolysis). Of all carriers, 42.4% were females (OR  = 1.24, 95% CI  = 0.49–3.15; p = 0.62). Carriage differed between the five villages ranging from 0% (Ndougou, n = 5 participants) to 45.7% (Village Tranquille, n = 46 participants, Table 1). There was an age-related carriage pattern with a peak colonization of 53.9% in participants between 10 and 20 years of age and a decreasing prevalence in subsequent age groups (Figure 1). No significant associations between *S. aureus* carriage and any recorded risk factor were detected (not shown).

Of the totality of 34 *S. aureus* isolates, 64.7% (n = 22) were susceptible to penicillin, *blaZ* PCR amplicons were only detected in penicillin resistant isolates (n = 12). In addition, 94.1% (n = 32) were susceptible to tetracycline and 88.2% (n = 30) to trimethoprim-sulfamethoxazole. All isolates were susceptible to oxacillin/methicillin, aminoglycosides, fluoroquinolones, macrolides, lincosamides (including inducible clindamycin resistance), nitrofurantoin, fosfomycin, rifampicin and vancomycin. Susceptibility to oxacillin/methicillin was further confirmed by the absence of *mecA*. Collecting and preparing medicinal herbs was not significantly related to a lower prevalence of antibiotic resistance (penicillin, tetracycline or trimethoprim-sulfamethoxazole) in colonizing *S. aureus* isolates (OR  = 0.23, 95% CI 0.02–1.51, p = 0.078).

### Virulence factors and *agr* groups

We detected 111 toxin encoding genes among 34 *S. aureus* isolates indicating a high prevalence of toxin co-possession. Table 2 shows the prevalence of the virulence factors tested and assigns these virulence factors to the total number of virulence genes, which were simultaneously detected. Overall, 73.5% (n = 25) of all isolates encoded one or more PTSAgs, with co-possession found in 15 isolates including 15 isolates (44%) that encoded the linked *seg-sei* loci. The *tst* gene was not detected but PVL-encoding genes were found in 55.9% (n = 19) of all isolates and were always co-detected with at least one other virulence determinant. The genes *seh* and *etd* were always co-detected with PVL-encoding genes (p = 0.238 and p = 0.113 respectively). Other virulence genes were only partially co-detected with PVL: *sea* (80.0%, p = 0.36), *seb* (14.3%, p = 0.03), *seg/sei* (60.0%, p = 0.67), *edin-B* (75.0%, p = 0.63). PVL was not co-detected with *sec* (p = 0.03) and *eta* (p = 0.44).

**Table 2:** Co-possession of virulence factor-encoding genes.

Toxin gene	Single possession	No. coexistent virulence factors (%)					Total,
		1	2	3	4	5	No. (%)
*lukS*-PV, *lukF*-PV (PVL)	0	2 (5.9)	6 (17.7)	7 (20.6)	3 (8.8)	1 (2.9)	19 (55.9)
*sea*	0	0	2 (5.9)	1 (2.9)	2 (5.9)	0	5 (14.7)
*seb*	0	4 (11.8)	0	2 (5.9)	1 (2.9)	0	7 (20.6)
*sec*	0	0	0	4 (11.8)	0	0	4 (11.8)
*seg-sei*	0	0	0	11 (32.4)	3 (8.8)	1 (2.9)	15 (44.1)
*seh*	0	0	3 (8.8)	0	0	0	3 (8.8)
*eta*	0	0	0	1 (2.9)	0	0	1 (2.9)
*etd*	0	0	1 (2.9)	2 (5.9)	0	1 (2.9)	4 (11.7)
*edin-B*	0	0	0	3 (8.8)	0	1 (2.9)	4 (11.7)
*hlg*	4 (11.8)	6 (17.7)	6 (17.7)	14 (41.2)	3 (8.8)	1 (2.9)	34 (100)

Among the accessory gene regulator subtypes, *agr* III was the most prevalent (61.76%, n = 21) followed by *agr* I and II (17.65%, n = 6 each) and *agr* IV (2.94%, n = 1, Table 3). Isolates encoding PVL-encoding genes were significantly less associated with *agr* I (OR  = 0; 95% CI: 0–0.52, p = 0.002) but often co-occurred with *agr* III (OR  = 3.1, 95% CI: 0.62–17.29, p = 0.107). *sea* was significantly associated with *agr* II (OR  = 41.12, 95% CI: 2.74–2679.92, p = 0.002). There was no significant association of all other virulence genes with any *agr* type.

**Table 3:** *agr* subtypes of *S. aureus* strains and number of co-occurring virulence-genes.

*agr* subtype	No. virulence factor encoding genes (%)					
	1	2	3	4	5	6
*agr* I (n = 6)	2 (33.3)	0	0	4 (66.7)	0	0
*agr* II (n = 6)	1 (16.7)	0	2 (33.3)	1 (16.7)	2 (33.3)	0
*agr* III (n = 21)	1 (4.8)	6 (28.6)	4 (19.1)	9 (42.9)	0	1 (4.8)
*agr* IV (n = 1)	0	0	0	0	1 (100)	0
Total	4	6	6	14	3	1

### Capsular polysaccharides

Detection of capsular polysaccharide (CP) encoding genes revealed a high prevalence of type 8 (CP8, 82.4%, n = 28) followed by type 5 (CP5, 14.7%, n = 5). One isolate was CP gene non-typable (2.9%). Interestingly, the PVL-encoding genes were co-detected with CP5 in 20% (OR  = 0.16, 95% CI 0–1.91, p = 0.146), and with CP8 in 64.3% (OR  = 8.44, 95% CI 0.79–447.45, p = 0.066). The heterogeneous distribution of CP was also reflected by a significant association of CP5 with *agr* I (OR  = 41.12, 95% CI 2.74–2679.92, p = 0.002) and CP8 with *agr* III (OR  =  infinity, 95% CI 2.6–infinity, p = 0.001).

### Genotyping

We identified 13 different *spa* types among 34 *S. aureus* isolates (Table 4). The most prevalent *spa* types were t1848 (23.5%, n = 8), t084, t148, t186 and t5941 (each 11.8%, n = 4). One participant carried two phenotypically different *S. aureus* isolates which had different *spa* types (t6025, t5941). Three *spa* types (t5941, t6020, t6025) have not been described before.

**Table 4:** Molecular characteristics of *S. aureus* isolates from Babongo Pygmies.

Sequence type (ST)	Clonal complex (CC)	MLST allelic profile	*spa* type	*agr* subtype	Capsular type (CP)	*lukS*-PV, *lukF*-PV (%)	No. isolates
1	1	1-1-1-1-1-1-1	t1931	III	8	+ (100)	2
1	1	1-1-1-1-1-1-1	t127	III	8	- (0)	1
5	5	1-4-1-4-12-1-10	t570	II	nt^a^	- (0)	1
5	5	1-4-1-4-12-1-10	t311	II	5	+ (100)	1
15	15	13-13-1-1-12-11-13	t084	II	8	+ (75%)	4
30	30	2-2-2-2-6-3-2	t1848	III	8	+ (100)	8
72	72	1-4-1-8-4-4-3	t148	I	5	- (0)	4
80	80	1-3-1-14-11-51-10	t5941	III	8	+ (100)	4
88	88	22-1-14-23-12-4-31	t186	III	8	- (0)	4
88	88	22-1-14-23-12-4-31	t6020	III	8	- (0)	1
121	121	6-5-6-2-7-14-5	t159	IV	8	+ (100)	1
188	188	3-1-1-8-1-1-1	t189	I	8	- (0)	2
1662	singleton	3-1-6-19-13-13-11	t6025	III	8	- (0)	1

a not typeable.

Ten different STs were found by MLST showing a Simpson's index of diversity (1-D) of 0.89 (Table 4). Among these, a hitherto unknown ST, designated ST1662 was detected. The most frequent ST was ST30 (23.5%, n = 8), exhibiting the following characteristics: t1848, *agr* III, CP8 and *luk*S-PV/*luk*F-PV-positive. PVL-encoding genes were found in isolates associated with *spa* types (ST) t1931 (ST1), t311 (ST5), t084 (ST15), t1848 (ST30), t5941 (ST80) and t159 (ST121, Table 4). STs did not cluster in distinct groups according to eBURST analysis. Interestingly, when comparing the STs of this study with the whole MLST database, all the STs of Babongo *S. aureus* isolates represented the predicted founders of their respective clonal complex, only the novel ST1662 was a singleton.

### Inter-village variation

Detailed inter-village comparison revealed demographic differences between the six camp-like villages (Table 1). The number of participants was imbalanced ranging from two in “Egouba” to 46 in “Village Tranquille” which is the biggest Babongo camp and the residence of the Babongo leader in the Ikobé region. Except for “Egouba”, resistance to penicillin was equally distributed among isolates from different camps. Resistance to tetracyline and trimethoprim-sulfamethoxazole was only found in “Village tranquille” (Table 1). There was no predominance of a single *spa* type or ST in a certain village. However, the following *spa* types were only found in one village: t084, t159, t311 (“Village Tranquille”), t570 (“Tsibanga”), t6020 (“Ossimba”) and t127, t6025 (“Soga”, Table 1).

## Discussion

To our knowledge, this is the first investigation of *S. aureus* isolates from a semi-nomadic indigenous African population. It provides a characterization of susceptibility to antimicrobial drugs, virulence factors and the clonal structure of the isolates. The main findings of our study are the high prevalence of PVL-positive isolates and the same genetic background of Babongo *S. aureus* isolates as pandemic clones.

Our survey needs to be considered as a representative population-based study, because it covers more than 30% of the Babongo population [21], has a balanced distribution of sex and shows a pagoda-shaped population pyramid typical of a developing community (Figure 1). Due to the semi-nomadic lifestyle of the participants, we cannot give the exact total number of the population in each village, but the total Pygmy population in the study area is estimated to be 300 persons [21]. The *S. aureus* carriage rate of 33% among Babongo Pygmies is similar to those reported worldwide ranging from 25 to 35% [22], [23]. Carriage corrected for age groups showed the highest colonization in teenagers (54%). This is comparable to the nasopharyngeal carriage rate reported from Europe showing a peak prevalence of over 50% at the age of ten years [24]. The absence of *S. aureus* in Ndougou is probably due to the small sample size of this village (n = 5, Table 1) and the higher mean age of the participants as carriage declines in older age groups (Figure 1).

Resistance to beta-lactams was rare. Only 35.3% of the isolates were resistant to penicillin, no MRSA was detected. This high prevalence of isolates susceptible to penicillin might be an indirect marker of a limited use of antibiotic agents in this population. However, community-associated MRSA (CA-MRSA) can also emerge in remote populations as shown for Australian Aborigines and North-American Indians [25]–[27]. The toxin gene profile differed clearly from European carrier isolates. Whereas we detected similar rates of *sea*, *sec*, *seh* and *eta*, the prevalence of genes encoding other superantigens and exfoliative toxins were higher in the Babongo *S. aureus* isolates compared to isolates originating from Europe: *seb* (20.6 vs. 3.8%), *etd* (11.7 vs. 5.2%) and *edin-B* (11.7 vs. 6.2%) [28]. Surprisingly, genes encoding SED-SEJ and TSST-1, which are common among carrier and clinical isolates in Europe (approx. 7–15% and 15–25% respectively) [14], [28], [29], were not detected in this Pygmy population.

The distribution of the capsule types was biased towards CP8 vs. CP5 (82.4% vs. 14.7%) compared to asymptomatic carriers in Europe (approx. 60–75% vs. 10–35%) [23], [30], [31]. CP5 and CP8 have been shown to impact the virulence of *S. aureus* and the clinical course of infection [32], [33]. Capsular polysaccharide expression is part of the *agr* regulon, we showed significant association of CP5 with *agr* subtype I (p = 0.002) and CP8 with *agr* subtype II (p = 0.001). This distribution of CP among *agr* subtypes has also been shown in isolates derived from bovine mastitis [34].

Interestingly, more than 55% of the *S. aureus* isolated from Babongo Pygmies carried PVL-encoding genes. This prevalence is comparatively high as only 1–2% of clinical methicillin-susceptible *S. aureus* (MSSA) isolates from Europe are PVL positive [13], [35]. PVL is a bacteriophage-encoded pore-forming toxin, which causes necrosis of tissues and has cytocidal effects on human neutrophils [1]. The clinical role of PVL is not yet fully understood and its role as a virulence factor remains controversial. PVL can be associated with necrotizing pneumonia in humans [36]. A rabbit model of necrotizing pneumonia has clearly demonstrated that PVL both activates polymorphnuclear leucocytes (PMNs) and macrophages and induces necrosis of PMNs [37]. Infected rabbits had the same clinical features of necrotizing pneumonia as described in humans, i. e. lung necrosis, edema, hemoptysis and death [37]. The high prevalence of PVL-positive *S. aureus* strains could therefore be a risk for Babongo Pygmies to develop necrotizing infections. However, studies with different animal models have shown conflicting results concerning the role of PVL. Some animal studies suggested PVL as major virulence factor in a mouse pneumonia model [38]. Other studies indicate that phenol soluble modulins might enhance the cytolytic effect of PVL [39] or have shown that α-hemolysin (α-toxin) or a point mutation in the *agr* P2 promoter are responsible for an increased virulence of PVL-positive strains in mice [40], [41]. In addition, other experiments did not find any evidence for PVL as a virulence factor in a murine model [42], [43]. However, it is known that PVL acts differentially on neutrophils of various species as PVL has a strong cytotoxic effect on human neutrophils but not on murine neutrophils [1]. Thus, the impact of a high prevalence of PVL-positive strains in a healthy Babongo population is still unclear. Further prospective studies are needed to analyze if PVL has an impact on the incidence of *S. aureus* infections in a neglected population.

Noteworthy, the presence of PVL-encoding genes was not associated with one distinct clonal lineage, but was distributed among different STs and *spa* types. A high prevalence of PVL could be a common feature of Sub-Saharan *S. aureus* isolates as high prevalence of PVL has been also found in non-pygmy populations from South Africa (100%), Mali (100% in *S. aureus* ST152), and Nigeria (42.7%) [3], [7], [44]. In contrast to our investigation, all but one of these studies included clinical isolates and might be therefore biased. However, high prevalence of PVL encoding genes is frequently found in pandemic CA-MRSA-clones and certain MSSA lineages (ST1, ST5, ST30, ST80) appear to be a reservoir of CA-MRSA [6]. In our study, we found very common STs (ST1, ST30, and ST121) amongst the Babongo Pygmies, some of them are pandemic clones [6], [45]. This is surprising as the Babongo Pygmies split apart from other humans at least 50,000 years ago and still live in isolated areas [46]. However, it is unclear, whether the same genetic background of Babongo *S. aureus* isolates and pandemic clones reflects the global spread of distinct clones or if it is the result of separate evolutionary processes in different geographic regions where the same successful clones were independently selected.

As shown for *Helicobacter pylori*, bacterial polymorphisms may reflect human phylogeography and historical migrations [47]. The genetic diversity in *H. pylori* decreases with geographic distance from East Africa mirroring the migration of its human host [10]. Interestingly, the Simpson's index of diversity among *S. aureus* STs from Babongo Pygmies was higher (0.89) than in a comparably remote Amerindian community in French Guiana (0.82), but was still lower than in urban communities in France, Algeria, Moldavia and Cambodia (0.92, 0.93, 0.92, 0.91) [8]. This is possibly due to a higher exchange and transmission between people from different regions and communities in the urban setting. Comparing genetic diversity of *S. aureus* isolates from isolated population may contribute to the discussion whether *S. aureus* shows a similar co-evolution and phylogeographical distribution patterns as observed for *H. pylori* [8], [10]. To confirm this, more population-based carrier studies are needed from different geographic regions to address possible factors of *S. aureus* transmission between a given isolated population and its neighboring communities.

One limitation of our study is the small sample size which might be only increased if those inhabitants who went hunting in the deep rain forest would have been included. Healthy male subsistence hunter might therefore be underrepresented. In addition, we failed to collect confident data about the use of antimicrobial drugs to analyze its impact on the carriage of resistant isolates. This limitation was due to a poor documentation of antibiotic treatments in the personal health care files and due to difficulty in recalling reliably the intake of antimicrobial agents. Another limitation of our study is the missing data about the incidence of *S. aureus*-related infections to assess the impact of the high prevalence of PVL on developing invasive disease. To record more confident data about the use of antibiotic drugs for each participant and to survey the incidence of *S. aureus* infections, future prospective studies are warranted.

In conclusion, our study provides the first insight in *S. aureus* isolates from an African Pygmy population. While we found a high prevalence of PVL-positive isolates, its impact on the incidence of *S. aureus* infection in remote populations is not clear yet. Many *S. aureus* isolates had the same genetic background as pandemic CA-MRSA clones raising the question of a common ancestor. We recommend a close surveillance of *S. aureus* isolates in remote indigenous African population to control the emergence of resistant isolates and to investigate the role of PVL-positive *S. aureus* isolates in neglected communities.

## Supporting Information

Checklist S1 STROBE checklist
(DOC)
