# Supplementary material for: Population Structure of Staphylococcus aureus from Remote African Babongo Pygmies
Source: PLoS Negl Trop Dis. 2011 May 10;5(5):e1150. doi: 10.1371/journal.pntd.0001150 (PMC3091839; doi:10.1371/journal.pntd.0001150)
Supplement: Checklist S1 — STROBE checklist (DOC) [file pntd.0001150.s001.doc]

STROBE Statement—checklist of items that should be included in reports of observational studies

|  | Item No | Recommendation |
| --- | --- | --- |
| **Title and abstract** | 1 | (*a*) Population Structure of Staphylococcus aureus from Remote African Babongo Pygmies (cross sectional study) |
|  |  | (*b*)  *Staphylococcus aureus* is a bacterium that colonizes humans worldwide. The anterior nares are its main ecological niche. Carriers of *S. aureus* are at a higher risk of developing invasive infections. Few reports indicated a different clonal structure and profile of virulence factors in *S. aureus* isolates from Sub-Saharan Africa. As there are no data about isolates from remote indigenous African populations, we conducted a cross-sectional survey of *S. aureus* nasal carriage in Gabonese Babongo Pygmies. The isolates were characterized regarding their susceptibility to antibiotic agents, possession of virulence factors and clonal lineage. While similar carriage rates were found like in populations of industrialized countries, isolates that encode the genes for the Panton-Valentine leukocidin (PVL) were clearly more prevalent than in European countries. Of interest, many methicillin-susceptible *S. aureus* isolates from Babongo Pygmies showed the same genetic background as pandemic methicillin-resistant *S. aureus* (MRSA) clones. We advocate a surveillance of *S. aureus* in neglected African populations to control the development of resistance to antibiotic drugs with particular respect to MRSA and to assess the impact of the high prevalence of PVL-positive isolates. |
| Introduction | | |
| Background/rationale | 2 | Population analysis of global methicillin-susceptible S. aureus (MSSA) isolates associated with PVL have recently indicated that PVL-positive MSSA and MRSA are phylogenetically related based on molecular epidemiological profiles and are dynamically interrelating. Moreover, it was shown, that PVL-positive MSSA are a likely reservoir for the development of PVL-positive MRSA via integration of Staphylococcus cassette chromosome mec (SCCmec) elements including the mecA gene conferring methicillin resistance. It is striking that reports from African countries have recently described a high prevalence of PVL-positive MSSA isolates in Nigeria and Mali and have supported the hypothesis that at least one common European MRSA clone associated with PVL (sequence type ST152 according to multilocus sequence typing (MLST)) could originate from African MSSA clones. Interestingly, a study on S. aureus colonization in Wayampi Amerindians in French Guiana revealed a predominance of ST1223 which is highly divergent from other global STs. Ruimy et al. hypothesize, that this association of highly divergent clones in an isolated remote population may reflect the co-evolution of humans and S. aureus as well as human migration |
| Objectives | 3 | We raise the question, whether the “out-of-Africa” hypothesis, as shown for Helicobacter pylori, might also be true for PVL-positive S. aureus clones now emerging across the globe. To address this question, we aimed to collect systematically S. aureus isolates independent from the healthcare setting, which is associated with the dissemination of isolates adapted to the specific selection pressure of the hospital environment. Therefore, we performed a cross-sectional S. aureus carrier study among the indigenous Pygmy population in Gabon. One to five percent of the Gabonese population is comprised by Pygmy hunter-gatherers. Almost 50% belong to the Babongo tribe, most of them are living in Waka National Parc, Central Gabon. |
| Methods | | |
| Study design | 4 | Cross-sectional survey of S. aureus carriage in asymptomatic Babongo-Pygmies, Gabon |
| Setting | 5 | The study took place in the Ikobé region, Central Gabon in November 2009. |
| Participants | 6 | *Cross-sectional study*—All Babongo or mixed Babongo-Bantu inhabitants of the Ikobé region were included if they provided a documented informed consent. Exclusion criteria were (i) infections of nostrils and (ii) a purulent rhinitis |
|  |  |  |
| Variables | 7 | Demographic data (self reported age, height, weight, sex and ethnic group) were recorded for each subject. Travel habits since birth and daily activities were recorded to assess risk factors for S. aureus carriage. Global positioning data of each village were taken by GPS-device (Garmin76 csx). |
| Data sources/ measurement | 8* | All demographic variables were self-reported by the study participant. Data about characteristics of S. aureus isolates were obtained by molecular analysis (PCR and sequencing) |
| Bias | 9 | We tried to include all members of the Babongo population in the Ikobè region to avoid any bias. As we were only able to enrol patients in the camps, young healthy hunters might be underrepresented as they usually spent the days in the deep forest. |
| Study size | 10 | We knew that about 300 inhabitants of the Ikobé region were Babongo and tried to enrol one third of the population. |
| Quantitative variables | 11 | Quantitativbe variables were not compared between groups as the study population was not divided in subgroups. |
| Statistical methods | 12 | (*a*) Proportions of categorical variables were tested using Chi-square test and Fisher’s exact test, where appropriate. Odds-ratio and the 95% confidence intervals were calculated to test for associations. The level of significance was α = 5%. All analyses were performed using the software “R” (http://cran.r-project.org, Version: 2.10.1) and package „epicalc“. |
|  |  | (*b*) Describe any methods used to examine subgroups and interactions- not applicable |
|  |  | (*c*) Explain how missing data were addressed- not applicable |
|  |  | (*d*)  *nal study*—If applicable, describe analytical methods taking account of sampling strategy- not applicable |
|  |  | (*e*) Describe any sensitivity analyses- not applicable |

Continued on next page

| Results | | |
| --- | --- | --- |
| Participants | 13* | (a) All villagers who had been in the village during our visit met the inclusion criteria (n = 103). |
|  |  | (b) Three persons from Egouba refused to participate |
|  |  | (c) Consider use of a flow diagram-Not applicable |
| Descriptive data | 14* | (a) The age distribution of participants showed a pagoda-shaped population pyramid with 45% of participants being ≤15 years old and 13% being ≥45 years old. Overall, 46% of participants were female. Of all participants, 65% have travelled at least to one of the nine capital cities of Gabon since birth. Twenty-six percent (n = 26) of the population had been hospitalized previously in dispensaries or primary-care hospitals. Females and males were equally distributed |
|  |  | (b) Indicate number of participants with missing data for each variable of interest_ not applicable |
|  |  | (c) *Cohort study*—Summarise follow-up time (eg, average and total amount)- not applicable |
| Outcome data | 15* |  |
|  |  |  |
|  |  | *Cross-sectional study—*Report numbers of outcome events or summary measures - not applicable |
| Main results | 16 | (*a*) Give unadjusted estimates and, if applicable, confounder-adjusted estimates and their precision (eg, 95% confidence interval). Make clear which confounders were adjusted for and why they were included- not applicable |
|  |  | (*b*) Report category boundaries when continuous variables were categorized- not applicable |
|  |  | (*c*) If relevant, consider translating estimates of relative risk into absolute risk for a meaningful time period- not applicable |
| Other analyses | 17 | The carriage rate was 33%. 64.7% (n = 22) were susceptible to penicillin, blaZ PCR amplicons were only detected in penicillin resistant isolates (n = 12). In addition, 94.1% (n = 32) were susceptible to tetracycline and 88.2% (n = 30) to trimethoprim-sulfamethoxazole. All isolates were susceptible to oxacillin/methicillin, aminoglycosides, fluoroquinolones, macrolides, lincosamides (including inducible clindamycin resistance), nitrofurantoin, fosfomycin, rifampicin and vancomycin. PVL-encoding genes were found in 55.9% (n = 19) of all isolates and were always co-detected with at least one other virulence determinant. he most prevalent spa types were t1848 (23.5%, n = 8), t084, t148, t186 and t5941 (each 11.8%, n = 4). PVL-encoding genes were found in isolates associated with spa types (ST) t1931 (ST1), t311 (ST5), t084 (ST15), t1848 (ST30), t5941 (ST80) and t159 (ST121). |
| Discussion | | |
| Key results | 18 | Our study is the first investigation of *S. aureus* isolates from a semi-nomadic indigenous African population. It provides a characterization of susceptibility to antimicrobial drugs, virulence factors and the clonal structure of the isolates. The main findings of our study are the high prevalence of PVL-positive isolates and the same genetic background of Babongo *S. aureus* isolates as pandemic clones. |
| Limitations | 19 | One limitation of our study is the small sample size which might be only increased if those inhabitants who went hunting in the deep rain forest would have been included. Healthy male subsistence hunter might therefore be underrepresented. We judge this bias to be small, as we study activities and inclusion also took place late in the evening when the hunters might have been back from the deeper forest. In addition, we failed to collect confident data about the use of antimicrobial drugs to analyze its impact of the carriage of resistant isolates. This limitation was due to a poor documentation of antibiotic treatments in the personal health care files and due to difficulty in recalling reliably the intake of antimicrobial agents. |
| Interpretation | 20 | While we found a high prevalence of PVL-positive isolates, its impact on the incidence of S. aureus infection in remote populations is not clear yet. Many S. aureus isolates had the same genetic background as pandemic CA-MRSA clones raising the question of a common ancestor |
| Generalisability | 21 | We recommend a close surveillance of S. aureus isolates in remote indigenous African population to control the emergence of resistant isolates and to investigate the role of PVL-positive S. aureus isolates in neglected remote communities. |
| Other information | | |
| Funding | 22 | The study was funded by the Deutsche Forschungsgemeinschaft (DFG) (EI 247/8-1, FR2569/2-1) and Bundesministerium für Bildung und Forschung (BMBF), Germany (01KI1009A "Skin Staph" [Susceptibility and Resistance towards Infections]). The funding institutions had no influence on the design, the performance and evaluation of the study. |

*Give information separately for cases and controls in case-control studies and, if applicable, for exposed and unexposed groups in cohort and cross-sectional studies.

**Note:** An Explanation and Elaboration article discusses each checklist item and gives methodological background and published examples of transparent reporting. The STROBE checklist is best used in conjunction with this article (freely available on the Web sites of PLoS Medicine at http://www.plosmedicine.org/, Annals of Internal Medicine at http://www.annals.org/, and Epidemiology at http://www.epidem.com/). Information on the STROBE Initiative is available at www.strobe-statement.org.
